# Changes in the effects of living with no siblings or living with grandparents on overweight and obesity in children: Results from a national cohort study in Japan

**DOI:** 10.1371/journal.pone.0175726

**Published:** 2017-04-17

**Authors:** Nayu Ikeda, Kana Fuse, Nobuo Nishi

**Affiliations:** 1 Center for International Collaboration and Partnership, National Institute of Health and Nutrition, National Institutes of Biomedical Innovation, Health and Nutrition, Tokyo, Japan; 2 Department of Information Collection and Analysis, National Institute of Population and Social Security Research, Tokyo, Japan; International Nutrition Inc, UNITED STATES

## Abstract

Effects of living without siblings and living with grandparents on overweight and obesity may change with child’s age. We aimed to examine these effects from early childhood to school age at the national level in Japan. Subjects were 43,046 children born in Japan during two weeks in 2001 who were followed annually from 2.5 to 13 years of age in the Longitudinal Survey of Newborns in the 21^st^ Century. We used measured body height and weight reported by participants at each survey and followed the criteria of the International Obesity Task Force to define overweight and obesity. Random-effects logit models by sex, adjusted for time-varying and time-invariant covariates, assessed odds ratios of overweight and obesity for living without siblings and for living with grandparents at each age. The likelihood of overweight and obesity was significantly higher at 8 years and older among children living without siblings, compared with those living with siblings, and odds ratios were highest at 11 years of age in boys (1.87, 95% confidence interval [CI]: 1.49, 2.33) and at 10 and 13 years of age in girls (1.75 [95% CI: 1.36, 2.23] and 1.73 [95% CI: 1.30, 2.31], respectively). It was also significantly higher at 5.5 years and older among children living with grandparents, compared with those living without grandparents, and odds ratios were highest at 10 and 13 years of age in boys (1.53 [95% CI: 1.30, 1.80] and 1.54 [95% CI: 1.27, 1.86], respectively) and at 11 years of age in girls (1.51, 95% CI: 1.24, 1.84). In Japan, living without siblings and living with grandparents may increase the likelihood of overweight and obesity at 8 and 5.5 years and older, respectively. Child’s age should be considered during formulation of strategies for prevention of overweight and obesity in these groups.

## Introduction

Prevention of childhood overweight and obesity is an important public health issue. Excess weight in children may negatively affect their lifetime health, increasing the risks of obesity in adulthood and consequent premature mortality and morbidity from non-communicable diseases [1]. Worldwide, the prevalence of childhood overweight and obesity increased in the late 20^th^ century [2, 3], and an estimated 41 million preschool children were overweight or obese in 2014 [2]. Japan follows the global trends: the prevalence of overweight and obesity in school-age children has increased in the last quarter of the 20^th^ century [4, 5]. Having gradually decreased since the 2000s, the prevalence of childhood overweight and obesity is still substantial in this country. For example, approximately 10% and 8% of 12-year-old boys and girls, respectively, were overweight or obese in 2015 [5]. To encourage healthy growth and lifestyles of children throughout the life course, the Japanese government has adopted a reduction in the prevalence of overweight and obesity in children as one of the targets in its national health promotion programs [6, 7].

Overweight and obesity in children result from a sustained excessive energy intake. This process is complex and involves factors related to the socioeconomic environment, including family [8, 9]. Family is an immediate environment for children and may have substantial control over their decisions on food choices and physical activity in daily life. Family structure therefore may be an important socioeconomic aspect to be considered in maintaining appropriate body weight of children.

Family structure of children in contemporary Japan has two major features. First, households with children most commonly have only one child. Of these households, the proportion with only one child has increased from 35% in 1986 to 46% in 2015, exceeding that of households having two children, which has decreased from 48% to 40% during this period [10]. Second, a substantial number of households with children also include grandparents, although the vast majority of households with children are nuclear families. Three-generation family households accounted for 16% of households with children in 2015, although the percentage has decreased from 27% in 1986 [10].

Previous studies have observed an increased likelihood of overweight and obesity among children living with no siblings [11–18] and children who have grandparents as informal caregivers or who live in three-generation families [19–24]. These children may become overweight and obese partly through being exposed to obesogenic behaviors and environments at home. For example, previous studies in Australia and Canada showed that, compared with children with siblings, those without siblings spent more time in low-intensity physical activity each day [25] and less time in moderate-to-vigorous-intensity physical activity [25, 26]. A previous study in the United States showed that, compared with children living in families with two or more children, only children were more likely to have a television in the child’s bedroom, spend over an hour in front of screen per day, and eat a meal infrequently with all the family members living in the household [27]. Regarding the effects of living with grandparents, a previous study in China showed that children mainly cared for by grandparents consumed unhealthful snacks and sugar-sweetened beverages more frequently compared with those mainly cared for by parents or other adults [21]. A previous study in Japan also showed that, compared with children mainly cared for by mothers, those mainly cared for by grandparents at age 3 years were more likely to eat between meals at age 6 and 12 years [23]. Moreover, the impacts of family structure on childhood overweight and obesity may change with age. As children grow up, some may get a younger sibling, or become more active socially and spend less time with grandparents at home. However, no previous study has assessed the impacts of living with no siblings or of living with grandparents on childhood overweight and obesity at the national level in Japan, nor is it well understood how these effects might change with age. The objective of this study therefore was to track the likelihoods of childhood overweight and obesity from living in a household with no siblings or with grandparents from early childhood to school age in Japan, using data from a nationally representative prospective survey.

## Materials and methods

### Ethics statement

This research was based on secondary analysis of observational survey data. Under the Statistics Act [28], the Ministry of Health, Labour and Welfare anonymized all individual-level data before providing the authors with the datasets for this study. No ethical review was sought based on the Ethical Guidelines for Medical and Health Research Involving Human Subjects [29], because this study used only information that had already been anonymized.

### Data sources

We used individual-level data from the Longitudinal Survey of Newborns in the 21st Century in Japan (2001 Cohort). Briefly, this is an observational cohort study of children born across Japan in 2001 [30]. The survey is carried out by the Ministry of Health, Labour and Welfare, and aims to obtain basic data for the planning and implementation of policies for the welfare of children and parents. Recruited through birth records from national vital registration systems, a total of 53,575 children born between January 10 and 17 or between July 10 and 17 in 2001 were eligible for the survey, and their families were first contacted on 1 August 2001 and 1 February 2002, respectively, when the children were 6 months old. The response rate was 87.8% (n = 47,015) for this initial survey. Participants were followed up annually on the same dates until age 5.5 years. After they entered primary school, the survey resumed at age 7 years on January 18 and July 18 for those born in January and July, respectively, and it continues on these dates every year. Response rates stayed around 90% across the follow-up surveys. A portion of the participants did not respond to all of the follow-up surveys, and the panel dataset were consequently unbalanced. Questionnaires were mailed to the home addresses of children. A parent or legal guardian was asked to complete the self-administered survey, and children aged 11 years or older were also asked to complete certain items.

The subjects of our analysis were the 43,754 children for whom a questionnaire was returned at least one of the 11 surveys conducted when they were 2.5–13 years old. After exclusion of participants who had missing data from birth records on birth weight (n = 11) and those who had missing or invalid data across all surveys on body height and weight (n = 697), the present analysis used a sample of 354,722 observations related to 43,046 children (183,966 observations related to 22,344 boys; 170,756 observations related to 20,702 girls).

### Statistical analysis

Using Stata 14 (StataCorp LP, College Station, TX, USA), we conducted all analyses separately by sex because of possible differences in the levels and trends in overweight and obesity between boys and girls. We used a random-effects logit model with the robust estimator of variance to calculate odds ratios of overweight and obesity by age for living with no siblings and for living with grandparents, adjusted for time-invariant and time-varying covariates. We accounted for within-person correlation over age by specifying an unstructured correlation matrix. Statistical significance was accepted at P <0.05.

The dependent variable in the model was a binary (overweight/obesity or not) repeated outcome across age. Each survey asked participants to report measured body weight and height to the nearest 0.1 kg and 0.1 cm, respectively, as well as date of measurement. We considered these measurements valid if they were taken within 180 days before or after a survey date. We calculated body mass index (BMI) as weight in kilograms divided by the square of height in meters. We considered a BMI value valid if it was ≥7 kg/m^2^ and ≤80 kg/m^2^. Following the criteria of the International Obesity Task Force, we considered children to be overweight or obese if they had a BMI equal to or greater than cut points by sex and age in months that corresponded to 25.0 kg/m^2^ at age 18 years [31].

To assess the rates of change in overweight and obesity by exposure over time, the model had interaction terms of the child’s age (reference group: age 2.5 years) with exposure at each age to living with no siblings and to living with grandparents. We created these exposure variables from self-reports on the relationship and number of individuals living with participants in the same household for the previous 3 months. We considered children as living with no siblings if they reported that they had no sibling living in the same household. We considered children as living with grandparents if they reported that they had any one of their paternal or maternal grandparents living in the same household.

To adjust for the effects of potential confounders, the model included time-invariant and time-varying covariates on sociodemographic characteristics and a behavioral mediating factor that might be relevant to childhood overweight and obesity. We carefully examined correlations between covariates to determine the final model. As time-invariant sociodemographic variables, we obtained birth order, birth weight, and maternal age from birth records attached to the survey data. We grouped birth order into first (reference group), second, and third and younger; birth weight in grams into <2,500 (reference group), 2,500–2,999, 3000–3,499, and ≥3,500; and maternal age in years into 15–24, 25–29 (reference group), 30–34, and ≥35. We also obtained self-reported educational attainment of fathers from the survey conducted at children’s age 1.5 years and grouped it into junior high school, high school, junior or career college, and university or higher education (reference group).

As for time-varying sociodemographic covariates, we used place of residence at each survey, grouped into eight geographical regions (Hokkaido, Tohoku, Kanto, Chubu, Kinki [reference group], Chugoku, Shikoku, and Kyushu) and overseas; urban/rural residence (12 major cities [reference group], other cities, towns/villages, overseas [omitted for the overlapping category in region of residence]); the number of hours worked outside the home by mothers per week, grouped into 0 (including students and the unemployed, reference group), <20, 20–39, and ≥40; and month in which anthropometric measurements were taken, grouped into April–September (reference group) and October–March. As a behavioral mediating factor, we used the number of hours of watching television per weekday, grouped into <1 (reference group), 1–1.9, 2–2.9, and ≥3. Data were missing for: hours worked outside the home by mothers per week at ages 5.5, 8, 9, 11, and 13 years and hours of watching television per weekday at age 13 years. We filled in missing values of these variables with immediately previous or immediately subsequent non-missing values.

After fitting the model, we computed time-varying, adjusted odds ratios of overweight and obesity for living with no siblings as an exponential of sum of coefficients on its time-invariant component and time-dependent deviations by age. We applied the same method in calculating time-varying, adjusted odds ratios of overweight and obesity for living with grandparents.

## Results

Table 1 summarizes distributions of time-invariant sociodemographic characteristics of the study subjects included in the analysis. Tables 2 and 3 show distributions of time-varying covariates on sociodemographic characteristics and a behavioral mediating factor used in this analysis in boys and girls, respectively, at selected ages. More than a third of both boys and girls were living without siblings at age 2.5 years. With the birth of younger siblings, the proportion fell below 15% by age 7 years and stayed almost constant thereafter. Approximately a fifth of boys and girls were living with grandparents at age 2.5 years, and the proportion remained virtually unchanged across the study period.

**Table 1:** Distribution (%) of time-invariant sociodemographic characteristics of the sample included in the analysis, by sex, in the Longitudinal Survey of Newborns in the 21^st^ Century, Japan.

Time-invariant covariates	Boys (N = 22,344)	Girls (N = 20,702)
Birth order		
First	48.3	49.5
Second	36.9	36.2
Third or younger	14.8	14.3
Birth weight (grams)		
<2,500	7.6	9.3
2,500–2,999	33.1	40.2
3,000–3,499	44.6	40.8
≥3,500	14.7	9.7
Maternal age (years)		
15–24	11.8	12.1
25–29	38.7	38.0
30–34	36.2	36.4
≥35	13.3	13.5
Educational background of fathers		
Junior high school	7.6	7.5
High school	38.0	37.9
Junior or career college	15.1	15.1
University or higher education	35.3	35.6
Miscellaneous/missing	4.0	4.0

**Table 2:** Distribution (%) of time-varying covariates on sociodemographic characteristics and a behavioral mediating factor used in the analysis in selected survey years, in boys, in the Longitudinal Survey of Newborns in the 21^st^ Century, Japan.

Time-varying covariates	Age at survey (years)					
	2.5	4.5	7	9	11	13
N	17,847	18,593	16,520	16,407	15,697	14,075
Living with no siblings	35.4	19.2	14.7	13.6	13.4	14.5
Living with grandparents	22.5	23.0	24.0	23.2	23.1	21.8
Region of residence*						
Hokkaido	3.6	3.4	3.4	3.4	3.5	3.4
Tohoku	7.6	7.3	7.3	7.4	7.3	7.4
Kanto	31.8	31.8	32.6	32.4	32.6	32.0
Chubu	18.5	18.9	18.6	18.7	18.9	19.3
Kinki	18.4	18.2	18.5	18.4	18.3	18.1
Chugoku	5.9	6.0	5.7	5.9	5.7	5.8
Shikoku	2.9	2.8	2.8	2.8	2.8	2.7
Kyushu	11.3	11.2	10.7	10.7	10.7	11.1
Overseas	0.2	0.4	0.3	0.3	0.3	0.2
Urban/rural of residence						
12 major cities	22.0	22.4	24.3	25.2	25.9	25.9
Other cities	58.7	64.3	65.6	65.3	64.8	64.9
Towns/villages	19.2	12.9	9.8	9.3	9.0	9.0
Overseas	0.2	0.4	0.3	0.3	0.3	0.2
Month of anthropometric measurement						
April–September	49.8	49.6	53.2	53.1	53.4	54.3
October–March	50.2	50.4	46.8	46.9	46.6	45.7
Hours worked by mothers per week						
0	63.4	52.5	44.0	42.8	32.7	25.2
<20	6.9	11.9	15.5	15.3	17.4	18.5
20–39	15.1	19.3	23.9	23.1	28.3	33.5
≥40	13.3	13.5	14.4	14.3	16.0	18.3
Missing	1.2	2.8	2.1	4.4	5.6	4.6
Hours of watching television per weekday						
<1	10.3	10.8	27.4	22.5	19.1	18.9
1–1.9	36.9	29.5	45.6	44.4	35.3	34.7
2–2.9	14.1	35.6	20.6	23.2	24.9	24.1
≥3	36.6	23.0	6.1	9.4	19.4	17.8
Missing	2.1	1.0	0.3	0.5	1.4	4.6

* Tohoku: Aomori, Iwate, Akita, Miyagi, Yamagata, Fukushima; Kanto: Ibaraki, Tochigi, Gunma, Saitama, Chiba, Tokyo, Kanagawa; Chubu: Yamanashi, Nagano, Niigata, Toyama, Ishikawa, Fukui, Shizuoka, Aichi, Gifu, Mie; Kinki: Shiga, Kyoto, Osaka, Hyogo, Nara, Wakayama; Chugoku: Tottori, Shimane, Okayama, Hiroshima, Yamaguchi; Shikoku: Kagawa, Ehime, Tokushima, Kochi; Kyushu: Fukuoka, Saga, Nagasaki, Oita, Miyazaki, Kumamoto, Kagoshima, Okinawa

**Table 3:** Distribution (%) of time-varying covariates on sociodemographic characteristics and a behavioral mediating factor used in the analysis in selected survey years, in girls, in the Longitudinal Survey of Newborns in the 21^st^ Century, Japan.

Time-varying covariates	Age at survey (years)					
	2.5	4.5	7	9	11	13
N	16,482	17,135	15,305	15,250	14,827	12,858
Living with no siblings	36.8	20.3	14.9	13.9	13.9	14.5
Living with grandparents	22.4	23.2	23.6	22.8	22.3	21.5
Region of residence*	22.4	23.2	23.6	22.8	22.3	21.5
Hokkaido	3.9	4.0	3.8	3.7	3.8	3.8
Tohoku	7.3	7.2	7.4	7.4	7.3	7.4
Kanto	32.4	32.1	33.0	32.8	32.9	32.2
Chubu	18.5	18.9	18.3	18.7	18.6	19.0
Kinki	17.7	17.6	17.7	17.5	17.9	17.6
Chugoku	6.1	6.1	5.9	6.0	6.0	6.1
Shikoku	3.2	3.1	3.2	3.1	3.1	3.2
Kyushu	10.8	10.7	10.4	10.4	10.2	10.3
Overseas	0.2	0.3	0.4	0.4	0.3	0.3
Urban/rural of residence						
12 major cities	22.1	22.3	24.4	25.2	25.9	25.4
Other cities	58.9	64.8	65.4	65.2	65.0	65.4
Towns/villages	18.8	12.6	9.8	9.2	8.9	8.9
Overseas	0.2	0.3	0.4	0.4	0.3	0.3
Month of anthropometric measurement						
April–September	50.9	50.6	51.8	51.7	52.4	54.8
October–March	49.1	49.4	48.2	48.3	47.6	45.2
Hours worked by mothers per week						
0	63.8	52.7	44.8	43.2	31.7	25.2
<20	7.2	11.6	15.8	15.7	18.3	18.4
20–39	14.7	19.3	23.1	22.5	27.8	32.6
≥40	13.2	13.6	14.4	14.2	16.3	19.4
Missing	1.1	2.8	1.9	4.5	5.9	4.4
Hours of watching television per weekday						
<1	10.2	12.6	29.6	23.1	19.4	19.6
1–1.9	36.9	30.3	43.8	42.7	31.9	31.8
2–2.9	14.1	34.2	20.0	23.8	24.7	22.8
≥3	36.9	21.9	6.2	10.1	22.9	21.7
Missing	1.9	1.0	0.3	0.3	1.1	4.1

* Tohoku: Aomori, Iwate, Akita, Miyagi, Yamagata, Fukushima; Kanto: Ibaraki, Tochigi, Gunma, Saitama, Chiba, Tokyo, Kanagawa; Chubu: Yamanashi, Nagano, Niigata, Toyama, Ishikawa, Fukui, Shizuoka, Aichi, Gifu, Mie; Kinki: Shiga, Kyoto, Osaka, Hyogo, Nara, Wakayama; Chugoku: Tottori, Shimane, Okayama, Hiroshima, Yamaguchi; Shikoku: Kagawa, Ehime, Tokushima, Kochi; Kyushu: Fukuoka, Saga, Nagasaki, Oita, Miyazaki, Kumamoto, Kagoshima, Okinawa.

**Fig 1:**
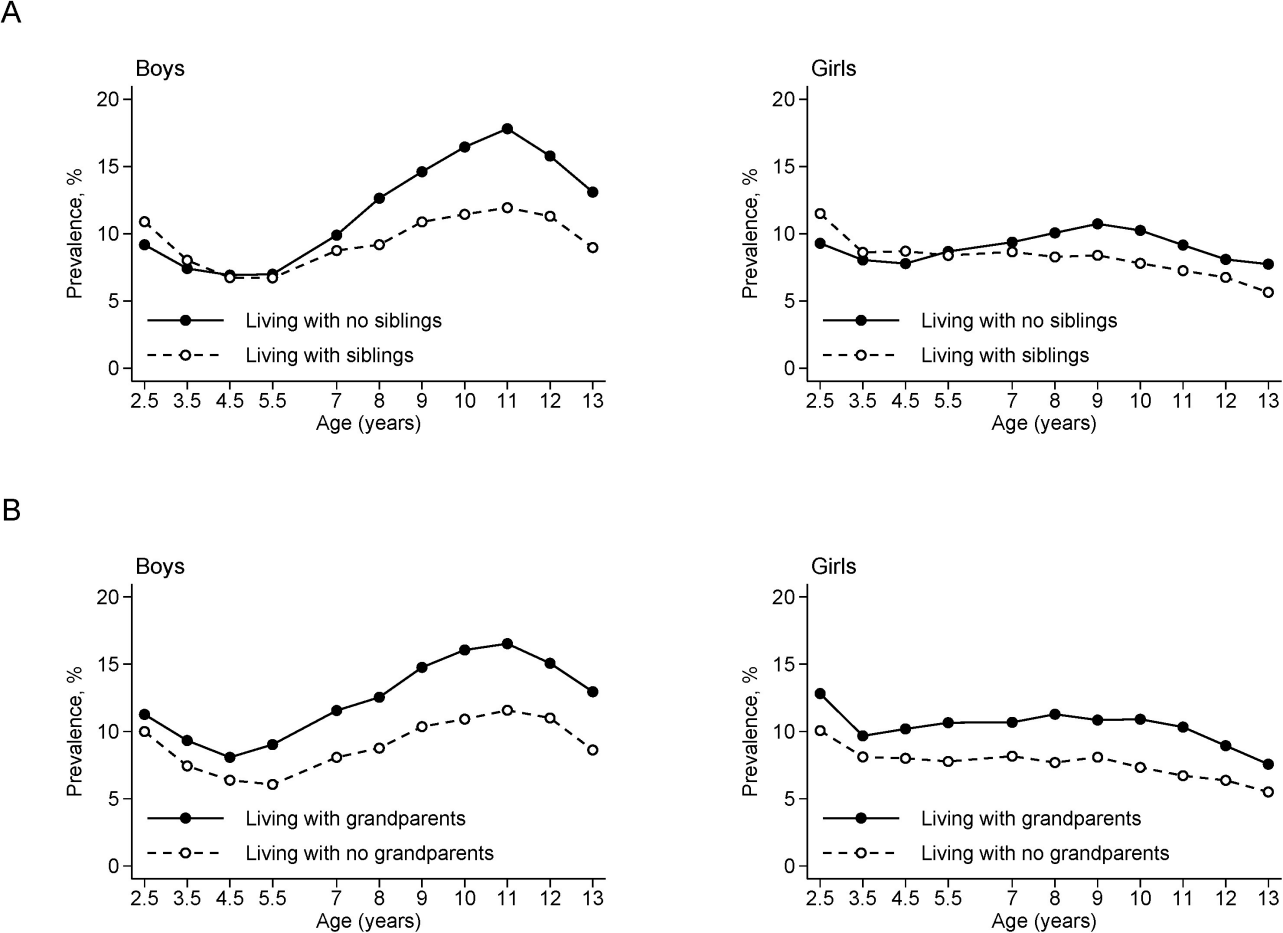
**National prevalence (%) of overweight and obesity among boys and girls between the ages of 2.5 and 13 years (2003–2014), by status of living with siblings (A) and with grandparents (B)**.

Fig 1 shows age trends in the prevalence of overweight and obesity by sex and status of living with siblings and grandparents. In both sexes, the prevalence of overweight and obesity was lower in participants living with no siblings at age 2.5 years compared with those living with siblings; however, this difference disappeared by age 5.5 years (Fig 1A). After participants entered primary school, for boys the prevalence increased until age 11 years in both groups, but boys living with no siblings had a larger increase compared with those living with siblings. For girls, the prevalence of overweight and obesity increased until age 9 years in those living with no siblings, while it consistently decreased across the study period in those living with siblings. In both sexes, the prevalence of overweight and obesity shifted with age almost in parallel between participants living with grandparents and those living with no grandparents (Fig 1B). The prevalence was persistently higher in participants living with grandparents compared with the other group across the study period, with the most notable differences of 40–50% and 30–50% observed in boys and girls, respectively, at ages 5.5–13 years.

Full results of odds ratios of overweight and obesity estimated from the random-effects logit model are provided in supporting information (S1 Table). Table 4 shows odds ratios of overweight and obesity for living with no siblings and living with grandparents, by age and sex, computed as an exponential of sum of coefficients on interactions with the child’s age and main effects after adjustment for confounders. Boys living with no siblings had significantly lower odds of being overweight and obese at ages 2.5–4.5 years compared with those living with siblings, while in girls these odds were not significantly different between the two groups before school age. Children living with no siblings had significantly higher odds of overweight and obesity compared with those living with siblings at 8 years and older in both sexes. The point estimates of odds ratios were highest at 11 years of age in boys and at 10 and 13 years in girls. Children living with grandparents had significantly higher odds of being overweight and obese, by approximately 30–50%, compared with those not living with grandparents at 5.5 years and older in both sexes. The point estimates of odds ratios were highest at 10 and 13 years of age in boys and 11 years of age in girls.

**Table 4:** Odds ratios (95% confidence intervals) of overweight and obesity for those living with no siblings and those living with grandparents, by age and sex†.

	Age(years)										
	2.5	3.5	4.5	5.5	7	8	9	10	11	12	13
Living with no siblings (Reference: living with siblings)											
Boys	0.73*	0.76*	0.79*	0.84	1.06	1.57**	1.47**	1.74**	1.87**	1.75**	1.72**
	(0.60, 0.90)	(0.62, 0.95)	(0.63, 1.00)	(0.66, 1.06)	(0.85, 1.33)	(1.26, 1.95)	(1.18, 1.83)	(1.40, 2.17)	(1.49, 2.33)	(1.39, 2.20)	(1.36, 2.18)
Girls	0.87	1.02	0.88	1.18	1.22	1.42*	1.63**	1.75**	1.52*	1.42*	1.73**
	(0.72, 1.06)	(0.83, 1.25)	(0.71, 1.10)	(0.94, 1.47)	(0.97, 1.55)	(1.13, 1.80)	(1.29, 2.06)	(1.36, 2.23)	(1.17, 1.97)	(1.08, 1.86)	(1.30, 2.31)
Living with grandparents (Reference: living with no grandparents)											
Boys	0.82	1.07	1.03	1.37*	1.36**	1.42**	1.45**	1.53**	1.47**	1.30*	1.54**
	(0.66, 1.03)	(0.88, 1.31)	(0.84, 1.26)	(1.13, 1.67)	(1.15, 1.62)	(1.20, 1.67)	(1.23, 1.71)	(1.30, 1.80)	(1.24, 1.73)	(1.09, 1.55)	(1.27, 1.86)
Girls	1.12	0.96	1.17	1.33*	1.13	1.45**	1.24*	1.44**	1.51**	1.38*	1.24
	(0.91, 1.38)	(0.78, 1.18)	(0.96, 1.42)	(1.10, 1.60)	(0.94, 1.36)	(1.21, 1.74)	(1.03, 1.49)	(1.19, 1.74)	(1.24, 1.84)	(1.11, 1.70)	(0.98, 1.58)

* P < 0.05

** P < 0.001.

† Covariates included in the models were sociodemographic characteristics (birth order, birth weight, maternal age at birth, educational attainment of father, region of residence, urban/rural of residence, number of hours worked by mothers per week, and month of anthropometric measurement) and a behavioral mediating factor (number of hours of watching television on weekdays).

## Discussion

Our study examined the impacts of living with no siblings and living with grandparents on childhood overweight and obesity in a national birth cohort survey in Japan with children between 2.5 and 13 years of age. To the best of our knowledge, this study is the first to assess and track changes in the effects of these two factors on weight status of children at the national level in Japan. Our results show that living with no siblings increased the likelihood of overweight and obesity consistently in school age, while these effects were not observed before school age. Living with grandparents had a similar effect to that of living with no siblings in school age; however, the effect was already apparent before school age.

Our results suggest that in Japan, living with no siblings is not a factor for overweight and obesity in early childhood, but that its effect increases and becomes pronounced in school age. This age trend may be partly attributable to the fact that some first-born children acquire siblings later. The estimated odds ratios in early childhood may subsequently be diluted by the presence of these children. In contrast, the marked excess risk in school age may reflect the genuine effects of remaining without a sibling. Our results were consistent with previous studies from cross-sectional analysis of local survey data, which showed increased risk of overweight and obesity in children having no siblings [11, 12, 15, 16, 18]. A cross-sectional analysis of data from a multi-center cohort study on children aged 2–9 years in eight European countries further showed that the excess risk of overweight and obesity from living with no siblings was larger for children at age 6–9 years compared with that at age 2–5 years [14]. Cross-sectional analyses of data from a national longitudinal survey in the United States showed that the excess risk of obesity among children living with no siblings increased between kindergarten and grade 8 [17].

Our results suggest that living with grandparents in the same household presents an increased likelihood of overweight and obesity in both boys and girls at age 5.5 years, a little earlier than the effect of living with no siblings. Given that most of the children living with grandparents stayed in this condition across the study period, the influence from grandparents may already be fixed to some extent in preschool age and continue through school age. Our results were consistent with previous local studies in China and Japan showing that children cared for by grandparents or living in three-generation families had an increased risk of childhood overweight and obesity [20, 21, 23, 24]. A previous study using data from a national longitudinal survey in the United Kingdom further showed that regardless of whether it was part-time or full-time, children between the age of 9 months and 3 years who were cared for by grandparents were more likely to be overweight and obese at age 3 years compared with those cared for by parents [19]. A previous study using data from a national birth cohort survey on children in urban low-income families in the United States showed that children who had lived with a grandmother at any time before age 3 years had an increased likelihood of overweight and obesity at age 3 years, although this effect was less pronounced at age 5 and 9 years [22].

Our study has certain limitations that should be considered in interpretation of its results. First, regarding identification of children living with no siblings, due to data unavailability we were unable to identify siblings living in a separate dwelling or to distinguish between biological and non-biological siblings. However, a cross-sectional study on low-income families in the United States suggests that additional effects of non-biological siblings on the results would be minor [18]. Second, our definition of living with grandparents was relatively simple. Information on how long children lived with grandparents in one year was not available from the survey. Moreover, in identifying children living with grandparents, we did not consider whether the children also lived with parents, i.e. whether they lived in multi-generational families or lived only with grandparents. However, additional effects of the presence of parents on study results would be negligible, because less than 1% of the survey participants lived without parents. Third, we did not separate the influence of living with a grandmother from that of living with a grandfather, because these two were strongly correlated with each other. Fourth, due to the lack of data in the survey, we were unable to control for confounding effects of important covariates such as maternal smoking during pregnancy, weight status of parents and grandparents, and any other genetic factors. Fifth, we examined only the direct relationships between the two factors of family structure and childhood overweight and obesity, without considering their effects on intermediate lifestyle factors such as diet and physical activity. Future studies with the national survey should explore how living with no siblings and living with grandparents influence children’s lifestyles.

In conclusion, over the life course of children from early childhood to school age, living with no siblings may increase the likelihood of overweight and obesity, particularly at school age, while the adverse effect of living with grandparents may appear shortly before school age. To effectively prevent childhood overweight and obesity, policymakers tasked with developing environmental strategies for improving lifestyles of children should consider such age-related changes in the influence of siblings and grandparents.

## Supporting information

S1 Table Full results of odds ratios of overweight and obesity in children between the ages of 2.5 and 13 years, by sex, estimated by the random-effect logit model from the Longitudinal Survey of Newborns in the 21st Century, Japan. (PDF)
