# Supplementary material for: Changes in the effects of living with no siblings or living with grandparents on overweight and obesity in children: Results from a national cohort study in Japan
Source: PLoS One. 2017 Apr 17;12(4):e0175726. doi: 10.1371/journal.pone.0175726 (PMC5393582; doi:10.1371/journal.pone.0175726)
Supplement: S1 Table — (PDF) [file pone.0175726.s001.pdf]

S1 Table. Full results of odds ratios of overweight and obesity in children between the ages of 2.5 and 13 years, by sex, estimated by the random-effect logit model from the Longitudinal Survey of Newborns in the 21st Century, Japan

Variables	Boys			Girls		
	Odds ratio	(95% CI)	P-value	Odds ratio	(95% CI)	P-value
Age (years)						
2.5	Reference			Reference		
3.5	0.55	(0.48, 0.63)	<0.001	0.64	(0.56, 0.74)	<0.001
4.5	0.41	(0.36, 0.48)	<0.001	0.63	(0.54, 0.72)	<0.001
5.5	0.39	(0.33, 0.45)	<0.001	0.59	(0.51, 0.69)	<0.001
7	0.67	(0.58, 0.78)	<0.001	0.71	(0.60, 0.83)	<0.001
8	0.72	(0.62, 0.84)	<0.001	0.61	(0.52, 0.71)	<0.001
9	1.00	(0.86, 1.16)	0.967	0.63	(0.54, 0.74)	<0.001
10	1.09	(0.94, 1.27)	0.271	0.51	(0.43, 0.60)	<0.001
11	1.17	(1.01, 1.36)	0.041	0.45	(0.38, 0.53)	<0.001
12	1.07	(0.92, 1.25)	0.369	0.39	(0.33, 0.46)	<0.001
13	0.66	(0.57, 0.78)	<0.001	0.31	(0.26, 0.37)	<0.001
Living with grandparents						
Living with no grandparents	Reference			Reference		
Living with grandparents	0.82	(0.66, 1.03)	0.082	1.12	(0.91, 1.38)	0.297
Living with siblings						
Living with siblings	Reference			Reference		
Living with no siblings	0.73	(0.60, 0.90)	0.003	0.87	(0.72, 1.06)	0.168
Interactions of age and living with grandparents						
Age 3.5 years and living with grandparents	1.30	(1.03, 1.65)	0.030	0.86	(0.67, 1.09)	0.210
Age 4.5 years and living with grandparents	1.25	(0.97, 1.61)	0.087	1.04	(0.81, 1.34)	0.741
Age 5.5 years and living with grandparents	1.67	(1.28, 2.17)	<0.001	1.19	(0.92, 1.54)	0.187
Age 7 years and living with grandparents	1.65	(1.27, 2.15)	<0.001	1.01	(0.77, 1.32)	0.927
Age 8 years and living with grandparents	1.72	(1.32, 2.23)	<0.001	1.30	(0.99, 1.69)	0.055
Age 9 years and living with grandparents	1.76	(1.36, 2.29)	<0.001	1.11	(0.85, 1.45)	0.454
Age 10 years and living with grandparents	1.85	(1.42, 2.42)	<0.001	1.28	(0.98, 1.69)	0.074
Age 11 years and living with grandparents	1.78	(1.36, 2.32)	<0.001	1.35	(1.02, 1.78)	0.033
Age 12 years and living with grandparents	1.58	(1.20, 2.07)	0.001	1.23	(0.92, 1.64)	0.155
Age 13 years and living with grandparents	1.87	(1.42, 2.47)	<0.001	1.11	(0.82, 1.51)	0.497
Interactions of age and living with no siblings						
Age 3.5 years and living with no siblings	1.04	(0.83, 1.32)	0.716	1.17	(0.93, 1.47)	0.190
Age 4.5 years and living with no siblings	1.09	(0.84, 1.40)	0.527	1.01	(0.79, 1.30)	0.927
Age 5.5 years and living with no siblings	1.15	(0.87, 1.52)	0.343	1.35	(1.03, 1.77)	0.028
Age 7 years and living with no siblings	1.45	(1.10, 1.92)	0.009	1.41	(1.06, 1.86)	0.018
Age 8 years and living with no siblings	2.14	(1.62, 2.82)	<0.001	1.63	(1.23, 2.17)	0.001
Age 9 years and living with no siblings	2.01	(1.53, 2.65)	<0.001	1.87	(1.40, 2.49)	<0.001
Age 10 years and living with no siblings	2.38	(1.80, 3.15)	<0.001	2.00	(1.49, 2.70)	<0.001
Age 11 years and living with no siblings	2.55	(1.92, 3.39)	<0.001	1.74	(1.28, 2.37)	<0.001
Age 12 years and living with no siblings	2.39	(1.78, 3.19)	<0.001	1.62	(1.18, 2.24)	0.003
Age 13 years and living with no siblings	2.36	(1.75, 3.16)	<0.001	1.99	(1.42, 2.78)	<0.001
Birth order						
First	Reference			Reference		
Second	0.98	(0.86, 1.10)	0.692	1.23	(1.08, 1.40)	0.002
Third or younger	1.00	(0.85, 1.18)	0.978	1.50	(1.26, 1.78)	<0.001
Birth weight						
<2500 grams	Reference			Reference		
2500–2999 grams	1.53	(1.23, 1.91)	<0.001	1.70	(1.36, 2.12)	<0.001
3000–3499 grams	2.15	(1.73, 2.67)	<0.001	2.94	(2.36, 3.66)	<0.001
≥3500 grams	4.20	(3.32, 5.31)	<0.001	5.96	(4.63, 7.67)	<0.001
Maternal age at birth						
15–24 years	1.11	(0.94, 1.32)	0.213	0.98	(0.82, 1.18)	0.832
25–29 years	Reference			Reference		
30–34 years	1.19	(1.06, 1.34)	0.003	0.99	(0.87, 1.12)	0.865
≥35 years	1.30	(1.11, 1.53)	0.001	1.18	(0.99, 1.40)	0.065
Educational background of fathers						
Junior high school	2.07	(1.70, 2.53)	<0.001	2.06	(1.67, 2.55)	<0.001
High school	1.53	(1.36, 1.72)	<0.001	1.45	(1.28, 1.65)	<0.001
Junior or career college	1.27	(1.09, 1.49)	0.002	1.18	(1.00, 1.39)	0.052
University or higher education	Reference			Reference		
Miscellaneous/missing	1.81	(1.38, 2.38)	<0.001	1.90	(1.42, 2.52)	<0.001

Variables	Boys			Girls		
	Odds ratio	(95% CI)	P-value	Odds ratio	(95% CI)	P-value
Region of residence						
Hokkaido	1.59	(1.20, 2.10)	0.001	1.35	(1.00, 1.81)	0.048
Tohoku	2.11	(1.70, 2.61)	<0.001	1.99	(1.59, 2.48)	<0.001
Kanto	1.37	(1.18, 1.58)	<0.001	1.30	(1.11, 1.52)	0.001
Chubu	1.11	(0.94, 1.32)	0.221	0.96	(0.80, 1.15)	0.628
Kinki	Reference			Reference		
Chugoku	1.14	(0.89, 1.46)	0.291	1.17	(0.91, 1.51)	0.211
Shikoku	1.58	(1.15, 2.16)	0.004	1.40	(1.02, 1.93)	0.036
Kyushu	1.38	(1.15, 1.66)	0.001	1.37	(1.12, 1.69)	0.003
Overseas	1.43	(0.73, 2.78)	0.297	2.04	(1.02, 4.09)	0.044
Urban/rural of residence						
12 major cities	Reference			Reference		
Other cities	0.99	(0.88, 1.11)	0.877	1.02	(0.90, 1.16)	0.736
Towns/villages	0.99	(0.83, 1.17)	0.866	1.27	(1.06, 1.52)	0.010
Overseas	Omitted			Omitted		
Month of anthropometric measurement						
April-September	Reference			Reference		
October-March	1.75	(1.63, 1.87)	<0.001	1.73	(1.61, 1.85)	<0.001
Working hours of mothers per week						
No work	Reference			Reference		
<20 hours	1.00	(0.91, 1.10)	0.945	1.00	(0.90, 1.12)	0.999
20-39 hours	1.05	(0.96, 1.14)	0.316	1.12	(1.01, 1.23)	0.028
≥40 hours	1.19	(1.07, 1.33)	0.002	1.44	(1.28, 1.62)	<0.001
Unknown/missing	1.22	(1.06, 1.41)	0.006	1.22	(1.03, 1.44)	0.019
Hours of watching television per weekday						
<1 hour	Reference			Reference		
1-1.9 hours	1.27	(1.18, 1.38)	<0.001	1.19	(1.09, 1.30)	<0.001
2-2.9 hours	1.42	(1.30, 1.56)	<0.001	1.37	(1.24, 1.52)	<0.001
≥3 hours	1.58	(1.42, 1.75)	<0.001	1.41	(1.26, 1.58)	<0.001
Missing	1.38	(1.10, 1.73)	0.006	1.27	(0.97, 1.66)	0.077
